# [Cu_3_(C_6_Se_6_)]*_n_*: The First Highly Conductive 2D π–d Conjugated Coordination Polymer Based on Benzenehexaselenolate

**DOI:** 10.1002/advs.201802235

**Published:** 2019-03-09

**Authors:** Yutao Cui, Jie Yan, Zhijun Chen, Jiajia Zhang, Ye Zou, Yimeng Sun, Wei Xu, Daoben Zhu

**Affiliations:** ^1^ Beijing National Laboratory for Molecular Sciences Key Laboratory of Organic Solids Institute of Chemistry Chinese Academy of Sciences Beijing 100190 China; ^2^ School of Chemistry Science University of Chinese Academy of Sciences Beijing 100049 China

**Keywords:** π–d conjugated coordination polymers, benzenehexaselenol, copper bis(diselenolene), electrical conduction

## Abstract

Nanocrystals of a 2D π–d conjugated copper bis(diselenolene) coordination polymer (Cu‐BHS, BHS = benzenehexaselenolate) are synthesized via a simple homogeneous reaction between cupric ions and benzenehexaselenol (H_6_BHS). Its 2D extended hexagonal lattice is confirmed by powder X‐ray diffraction, and further characterized by scanning electron microscopy, transmission electron microscopy, and X‐ray photoelectron spectroscopy. The electrical conductivity measured on compressed powder sample reaches 110 S cm^−1^ at 300 K, which is among the highest value ever reported for coordination polymers. Furthermore, the intrinsic metallic characteristics of Cu‐BHS are confirmed by ultraviolet photoelectron spectroscopy and band structure calculation.

2D materials, represented by graphene, have received great research interests because of their exceptional physical properties and promising applications.^1^, ^2^ Recently, there are growing interests in design and synthesis of 2D covalent or noncovalent organic materials with in‐plane π‐conjugation structure. In the past five years, 2D coordination polymers (CPs) constructed from metal ions with square‐planar coordination geometry and benzene‐ or triphenylene‐derived ligands with orthodisubstituted N, O, or S donor atoms exhibit highly electrical conductivity,^3^, ^4^, ^5^, ^6^, ^7^, ^8^, ^9^, ^10^, ^11^, ^12^, ^13^, ^14^, ^15^, ^16^ which were found to be promising active electrodes in electrocatalysis, transparent electrodes for photovoltaic solar cells, chemoreceptive sensors, and supercapacitors.^17^, ^18^, ^19^, ^20^, ^21^, ^22^, ^23^, ^24^, ^25^, ^26^, ^27^, ^28^, ^29^ The planar structure of these graphene analogues facilitates the charge carriers transport through bonds, enabling the materials in this class be good candidates in electronics. Of which, Cu‐BHT (BHT = benzenehexathiol) possesses the highest value of electrical conductivity and a unique structure. As shown in Figure S1 (Supporting Information), in the structure of Cu‐BHT, one BHT connects with other six BHTs through the shared Cu atoms in a square‐planar manner, resulting in a dense 2D structural topology without obvious pores, and with a continuous 2D Cu—S network.^7^ In other reported materials of this class, each organic ligand connects with other three ligands through the shared square‐planar coordinated late‐transition‐metal nodes, forming a porous 2D extended honeycomb structure.^30^ The special structure of Cu‐BHT and the continuous Cu—S network might be the basic reason of the high electrical conductivity and the metallically conductive behavior. Furthermore, the existence of bulk superconductivity with *T*_c_ = 0.25 K has been established in Cu‐BHT films with improved crystallinity very recently.^31^ Inspired by this exciting result, we expect to explore new CPs with similar structure and high electrical conductivity, which will pave a new way for searching molecular superconductors.

Heavy‐atom substitution is a successful strategy for constructing organic superconductors based on charge‐transfer salt.^32^ The organic molecule benzenehexaselenol (H_6_BHS), an analogue of BHT with all six S atoms substituted by heavier Se atoms, is an ideal ligand. Here we report a 2D π–d conjugated copper bis(diselenolene) CP Cu‐BHS containing BHS as bridge linker, which possesses a structure similar to that of Cu‐BHT as well as excellent electrical transport character.

To the best of our knowledge, the synthesis of BHS has not been reported yet, indicating the big challenge of the preparation of H_6_BHS. The synthesis of protected BHS hexakis(tert‐butylseleno)benzene (*t*‐Bu_6_BHS) was reported by Turner and Vaid in 2012.^33^ As the steric congestion of six selenolate anions on Na_6_C_6_Se_6_ leads to its instability, the reductive cleavage of the protective tertiary butyl groups under Birch conditions using sodium in anhydrous liquid ammonia was not successful.^33^ An alternative approach is Lewis acid promoted deprotection protocol. A method employing BBr_3_ for the synthesis of ortho‐benzene‐polythiols has been reported.^34^ And the resulting product dithiaboroles were also successfully used for the metal bis(dithiolene) complexes production. Moreover, we introduced the Lewis acid induced deprotection procedure to the synthesis of benzene‐ and triphenylene‐polyselenols and CPs containing these ligands (relevant results are in preparation). The method of boron tribromide as Lewis acid to promote the reproduction of the alkyl benzene‐polyselenols was used for the production of ligand H_6_BHS and coordination polymer based on this ligand. The intermediate diselenaboroles BHS(BBr)_3_ can be separated and utilized as a precursor reagent for the production of metal bis(diselenolene) CPs. When BHS(BBr)_3_ was dispersed in alcohol solvent, the hydroxyl makes the Se—B bond rupture and leads to the formation of the ligand H_6_BHS in situ (as illustrated in **Figure** **1**).

**Figure 1:**
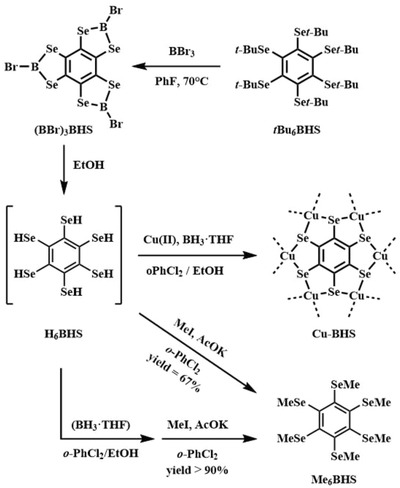
Synthesis of Cu‐BHS and Me_6_BHS.

To determine the formation of the ligand H_6_BHS, experimental verification was conducted. Under the same condition that we used in the synthetic procedure of Cu‐BHS, the ligand H_6_BHS was generated in situ from BHS(BBr)_3_ in mix‐solvent of ethyl alcohol and *o*‐dichlorobenzene. Then the alcohol solvent with low boiling point was evaporated under reduced pressure. Methylation of BHS occurred in the presence of iodomethane and potassium acetate. 1,2,3,4,5,6‐hexa(methylselanyl)benzene (Me_6_BHS) was obtained in the yield of 67% over three steps. When BH_3_•tetrahydrofuran (THF) was introduced to the synthetic procedure, the total yield of Me_6_BHS increase to more than 90%. Moreover, the nuclear magnetic resonance results (Figures S14–S16, Supporting Information) measured on the crude products show little peaks of by‐product. These results verify the formation of H_6_BHS with high yield and purity. And the addition of BH_3_•THF is indeed beneficial to increase the yield of Me_6_BHS, which indicate possibly positive effect of BH_3_ in the synthetic protocol of Cu‐BHS.

The coordination polymer Cu‐BHS was synthesized via simple homogeneous reaction of BHS formed in situ and cupric salt in a degassed mixed solvent of ethyl alcohol and *o*‐dichlorobenzene, and in the presence of BH_3_•THF. The bulk samples of Cu‐BHS were synthesized for more than five times, and with good repeatability and consistency. The synthesis of film sample via the previously reported interfacial reactions was not successful. The reason might be the poor solubility and stability of H_6_BHS.

The powder X‐ray diffraction (PXRD) result (**Figure** **2**a) shows good crystallinity of the Cu‐BHS powder. Considering the similar molecular structure of BHS and BHT, and the first intensive diffraction peak appears at the position close to that of Cu‐BHT complex, we proposed a 2D hexagonal lattice as [Cu_3_C_6_Se_6_]*_n_* according to what we found in Cu‐BHT complex. As shown in Figure 2b, in the 2D fully filled honeycomb model, one BHS connects with other six BHSs through the shared square planar coordinated Cu atoms and each Se atom chelates with two Cu atoms. A continuous 2D Cu—Se network could be observed in the dense lattice without obvious pores. A comparison of the experimental data with the simulated 3D crystals of AA, AB, and slipping AA stacking patterns (Figure S2, Supporting Information) reveals that both the positions and intensity profile of the PXRD calculated from the slipping AA stacking (cell parameter: *a* = 15.323, *b* = 8.968, *c* = 3.681 Å, α = 90, β = 92.68, γ = 90° in *C*2/*m* space group) are consistent with the experimental result of Cu‐BHS. Pawley refinement also shows that the PXRD pattern can be well reproduced with this structure model, as negligible difference can be observed (Figure 2a). The prominent peaks at 2θ = 11.6° and 20.2° indicate a long‐range order within the *ab* plane. The broadness of diffraction peak at 2θ = ≈25°, corresponding to the [001] reflections, might attribute to low dimensions along *c*‐axis in the crystallites.

**Figure 2:**
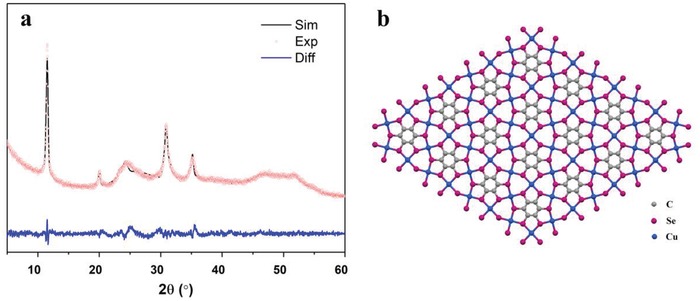
a) PXRD patterns of Cu‐BHS (red circle), the Pawley refinement result (black curve), and their difference (blue curve) and b) the 2D lattice of Cu‐BHS.

The component of Cu‐BHS was analyzed via elements analysis and inductively coupled plasma optical emission spectrometer. The contents of carbon, copper, and selenium were ascertained to be 9.91%, 26.59%, and 63.03%, in accordance with the calculated results (9.79%, 25.89%, and 64.33%) according to the formula of [Cu_3_C_6_Se_6_]*_n_*.

The X‐ray photoelectron spectroscopy full spectrums of Cu‐BHS (Figure S5, Supporting Information) detected C, Se, and Cu resonance peaks and verified the absence of B, Br, and F which were contained in the starting materials. In the Cu 2p region (Figure S6a, Supporting Information), two peaks at the binding energies of ≈929 and ≈948 eV correspond to Cu 2p_3/2_ and Cu 2p_1/2_. No obvious shake‐up satellite peak reveals the absence of Cu(II), indicating charge transfer between organic ligands and Cu ions and suggesting strong π–d interaction among the 2D latice of Cu‐BHS. Two peaks located at 928.8 and 930.3 eV can be discerned in the Cu 2p_3/2_ peak, which indicates two kinds of Cu exist with different chemistry environments. The weaker peak may originate from Cu ions at the edge of the Cu‐BHS layers or the surface of the nanocrystals.^7^ The type and location of these peaks are similar with that of Cu‐BHT and reported Cu‐bis(diselenolene) CPs.^7^, ^35^ In the Se 3d region (Figure S6b, Supporting Information), two peaks at the binding energies of 55.1 and 56.0 eV are observed for 3d_5/2_ and 3d_3/2_, in agreement with those of previously reported metal‐diselenolene CPs.^35^, ^36^

The morphology characteristics of Cu‐BHS materials were investigated by scanning electron microscope (SEM) and transmission electron microscope (TEM). Only one morphology was observed. As shown in **Figure** **3**, Cu‐BHS powder is composed of flaky nanocrystals with the size varying from several tens to more than one hundred of nanometers in width and several hundreds of nanometers in length. The nanocrystals of Cu‐BHS are clean and homogeneous. And the elemental mapping of single nanocrystal (Figure S4, Supporting Information) shows homogeneous distribution of C, Cu, and Se, respectively.

**Figure 3:**
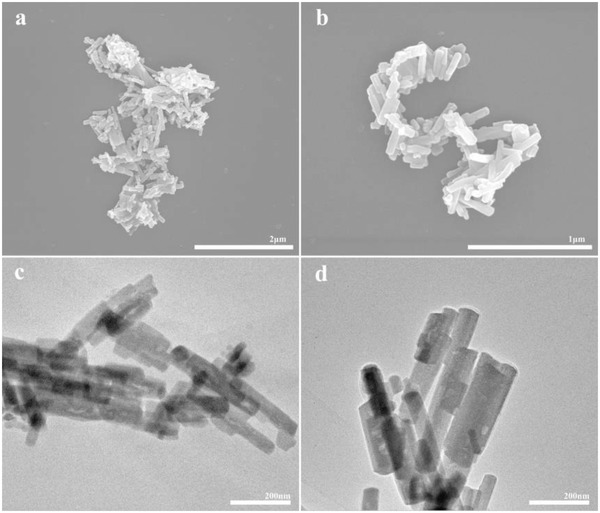
a,b) SEM and c,d) TEM images of Cu‐BHS.

The electronic properties of bulk Cu‐BHS sample were investigated. The electrical conductivity (σ) of Cu‐BHS was obtained from a standard four‐probe measurement of pressed pellet, which displays conductivity of 110 S cm^−1^ at 300 K. This value is of same order as Cu‐BHT (pellet, σ = ≈200 S cm^−1^ at 300 K).^20^ To the best of our knowledge, the σ of Cu‐BHS is one of the highest values of CPs measured on pressed pellet.^30^, ^37^ Temperature dependence of electrical conductivity was also investigated. As presented in **Figure** **4**a, the σ of Cu‐BHS increases almost linearly with the heating temperature from 10 to 400 K and is reversible upon cooling. The temperature dependence of σ is a typical behavior of semiconductors. It is noteworthy that the σ‐T dependence is weak (σ_400K_/σ_10K_ = 1.92). Because of the existence of large quantities of grain boundaries between crystallites in the pellet samples, the temperature dependence of σ obtained upon the pressed pellets does not accurately reflect the intrinsic charge transport properties of [Cu_3_BHS]*_n_*. The thermally activated electric conductive behavior in pellets might be dominated by the intergrain hopping transport of charge carriers. Similar thermally activated conductive behavior was also detected in Cu‐BHT with intrinsically metallic nature.^7^ Infrared spectra (IR) of Cu‐BHS (Figure S9, Supporting Information) shows strong continuous absorption peaks in all of the maximal region (2500–25 000 nm) what the IR spectrometer reaches. The result indicates a gapless nature of Cu‐BHS.

**Figure 4:**
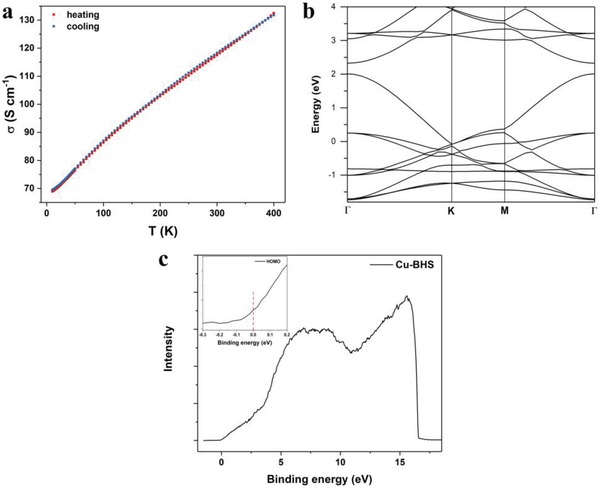
a) Temperature dependence of electrical conductivity measurement of Cu‐BHS pellet. b) Band structure of the monolayer of Cu‐BHS complex calculated at PBE level. K point *Γ* = (0,0,0); K = (−0.333,0.667,0.000); M = (0.000,0.500,0.000). The Fermi level is at zero. c) UPS of Cu‐BHS and the inset is detailed UPS of the Fermi edge.

To further understand the electrical transport properties of this coordination polymer, band structure of the single layer of Cu_3_C_6_Se_6_ lattice was calculated. As shown in Figure 4b, there are highly dispersive bands crossed by the Fermi level similar to that of the Cu‐BHT complex, showing the intrinsic metallic nature of this material. In the 3D lattice, due to the interlayer electronic coupling, the bands are expected to be broader, and the metallic character will not change (Figure S8, Supporting Information). The electronic structure of Cu‐BHS was also further characterized by ultraviolet photoelectron spectroscopy (UPS) (Figure 4c). Fermi edges revealed on the UPS indicate the electronic bands cross the Fermi level, which is coincident with the highly conducting behavior observed in electrical conductivity measurement and shows the metallic nature of Cu‐BHS.

In conclusion, the benzenehexaselenol (H_6_BHS) and a 2D copper bis(diselenolene) CPs containing BHS as linking bridge were synthesized for the first time. The benzenehexaselenol ligand was prepared in situ after alcoholysis of benzenehexaselanyl derived diselenaborole precursor. Nanocrystals of Cu‐BHS were prepared via a simple homogeneous reaction. The structure of Cu‐BHS is a Cu‐BHT like fully filled 2D honeycomb topology with continuous Cu—Se network. The electrical conductivity of Cu‐BHS measured on pressed pellet reaches 110 S cm^−1^ at 300 K, and the band structure calculation and UPS result confirm the intrinsically metallic characteristics. Based on the present results, further research on fundamental physical properties and advanced electronic application are worth pursuing.

## Conflict of Interest

The authors declare no conflict of interest.

## Supporting information

Supplementary
