# Supplementary material for: [Cu3(C6Se6)]n: The First Highly Conductive 2D π–d Conjugated Coordination Polymer Based on Benzenehexaselenolate
Source: Adv Sci (Weinh). 2019 Mar 9;6(9):1802235. doi: 10.1002/advs.201802235 (PMC6498113; doi:10.1002/advs.201802235)
Supplement: Supplementary file 1 — Supplementary [file ADVS-6-1802235-s001.pdf]

Copyright WILEY-VCH Verlag GmbH & Co. KGaA, 69469 Weinheim, Germany, 2019.

ADVANCED SCIENCE
Open Access

Supporting Information

for *Adv. Sci.,* DOI: 10.1002/advs.201802235

$[Cu_3(C_6Se_6)]_n$: The First Highly Conductive 2D π–d Conjugated Coordination Polymer Based on Benzenehexaselenolate

Yutao Cui, Jie Yan, Zhijun Chen, Jiajia Zhang, Ye Zou, Yimeng Sun, Wei Xu, and Daoben Zhu**

Supporting Information

$[Cu_3(C_6Se_6)]_n$: The first highly conductive two-dimensional π-d conjugated coordination polymer based on benzenehexaselenolate

Yutao Cui, Jie Yan, Zhijun Chen, Jiajia Zhang, Ye Zou, Yimeng Sun, Wei Xu*, and Daoben Zhu*

Experimental section:

General remarks

The protected ligand t-Bu_6BHS was synthesized by a slightly modified literature method.[33] The NMR results of t-Bu_6BHS are consistent with the reported literature (**Figure S13**). Ethyl alcohol, o-dichlorobenzene, AcOK, and $Cu(BF_4)_2$•$6H_2O$ were purchased from Acros Organics. All the solvents were degassed with freeze-pump-thaw cycling before using. Boron tribromide, fluorobenzene and iodomethane were purchased from J&K and used as received.

Synthesis

BHS(BBr)$_3$: Under argon atmosphere, t-Bu_6BHS (888mg, 1mmol) was dissolved in fluorobenzene (60ml), then BBr_3 (1.53g, 6mmol) was added. The stirred resulting mixture was heated at 70°C for 12h. During which, a lightly yellow precipitate formed. Then the reaction mixture was cooled to rt. The precipitate was filtered off and washed with hexane. The solid production was used for the next reaction as soon as possible and without further treatment. As the poor solubility and sensitive B-Br bond, it's difficulty to do characterization of BHS(BBr)$_3$. And the attempt at dissolving BHS(BBr)$_3$ in sodium hydroxide aqueous solution to get a BHSNa$_6$ solution leaded to the decomposition of BHS as description in literature. But we found that dissolving BHS(BBr)$_3$ in a degassed solution of AcONa-d3 in MeOH-d4 (5% w/w) produce a lightly yellow solution, which is stabilized under argon atmosphere for hours. The ^{13}C -NMR spectra generated by using this solution shows a distinct peak at 136.5 ppm, which is corresponding to the signal of sp2 carbon in BHS fragment (**Figure S12**).

Cu-BHS: Under argon atmosphere, freshly prepared BHS(BBr)$_3$ (1mmol) was dispersed in degassed ethyl alcohol (60ml) by ultrasonic for about 30 seconds. Then degassed o-dichlorobenzene (60ml) followed by $Cu(BF_4)_2$•$6H_2O$ (1.04g, 3mmol) was added to the stirred

mixture and a dark blue precipitate formed immediately. The reaction mixture was stirred for 0.5h and a solution of BH_3•THF in THF (1.0M, 6ml) was added. The resulting mixture was stirred at rt for another 12h. The precipitate was filtered off and washed with distilled water, methanol, and ether thoroughly. The final product **Cu-BHS** was obtained as mineral blue powder after drying under vacuum at rt for 12 hours. Anal. Calcd for $C_6Se_6Cu_3$: C, 9.79; H, 0.00; N, 0.00; Cu. 25.89; Se, 64.33. Found: C, 9.91; H, <0.30; N, <0.30; Cu, 26.59; Se, 63.03.

Me_6BHS, method 1: Under argon atmosphere, freshly prepared BHS$(BBr)_3$ (synthesized from 1mmol of t-Bu_6BHS) was dispersed in degassed ethyl alcohol (30ml) by ultrasonic for about 30 seconds followed by the addition of degassed o-dichlorobenzene (30ml). Then alcohol solvent was removed in reduced pressure. AcOK (981mg, 10mmol) and iodomethane (2.28g, 1ml, 16mmol) were added and the resulting mixture was stirred at rt for 12h. The reaction solvent was diluted by 50ml of DCM and washed with water and saturated NaCl aqueous solutions. The organic phase was collected and dried by $MgSO_4$. The organic solvents were removed under reduced pressure and the residue was purified by column chromatography (silica gel 200-300 mesh, n-Hexane:DCM=2:1). The final product was obtained as yellow powder after drying under vacuum at rt for 12 hours (425mg, yield=67%). ^{1}H NMR (400 MHz, $CDCl_3$) δ 2.43 (s, 18H). ^{13}C NMR (400 MHz, $CDCl_3$) δ 144.24, 77.48, 77.16, 76.84, 14.19. HRMS (m/z): $[M]^+$ calcd. for $C_{12}H_{18}Se_6^+$ 637.64156; found 637.64374.

Me_6BHS, method 2: According **method 1**, a solution of BH3•THF in THF (1.0M, 6ml) was added after the addition of o-dichlorobenzene. The reaction mixture was stirred for 0.5h before the alcohol solvent was removed in reduced pressure. The final product was obtained following the synthetic process as **method 1** (630mg, yield>90%). ^{1}H NMR (400 MHz, $CDCl_3$) δ 2.43 (s, 18H). ^{13}C NMR (400 MHz, $CDCl_3$) δ 144.24, 77.48, 77.16, 76.84, 14.18. HRMS (m/z): $[M]^+$ calcd. for $C_{12}H_{18}Se_6^+$ 637.64156; found 637.64195. Anal. Calcd for $C_{12}H_{18}Se_6$: C, 22.66; H, 2.85; N, 0.00. Found: C, 22.81; H, 2.63; N, <0.30.

Characterization

The content of C, H and N was analyzed by a Flash EA 1112 (Thermo Fisher Scientific). The content of Cu, and Se was carried on inductively coupled plasma optical emission spectrometer (Optima 5300DV, Perkin Elmer). The sample for ICP-oes measurement was prepared by dissolving them into heated and stirred fuming nitric acid. Then the resulting solution was diluted to a known volume by distilled water. ^{13}C-NMR spectra of t-Bu_6BHS

was recorded at 500 MHz (Bruker) and other NMR spectra was recorded at 400 MHz (Bruker). The chemical shifts were reported in parts per million (ppm) using the residual solvent signals as internal standards. Powder X-ray diffraction (PXRD) patterns were obtained at a PANalytical Empyrean II X-Ray diffractometer using Cu Ka irradiation (λ=1.5406 Å). XPS and UPS were performed by using AXIS Ultra-DLD ultrahigh vacuum photoemission spectroscopy system (Kratos Co.). A monochromatic magnesium Ka source (1253.8 eV) and a He I source (21.11 eV) were used for XPS and UPS, respectively. And Cu-BHS powder and a pressed pellet (thickness = ~0.05mm) were used for XPS and UPS measurements, respectively. The IR spectrum of Cu-BHS was obtained at a TENSOR-27 spectrometer (Bruker). Thermogravimetric analysis (TGA) was performed on a PerkinElmer TGA 8000 instrument under nitrogen atmosphere. The materials were compressed into cuboid pellets (5mm×10mm×~0.1mm) under a pressure of 10 MPa to measure the electrical conductivity. The temperature-dependent electrical conductivity was measured using four-contact probe method on the pressed pellets (under the electric current of 10mA) by using a KEITHLEY 2002 Multimeter (Keithley Instrument Inc.).

Pawley refinement was carried out with crystal structures derived from that of Cu-BHT. Reflex, a software package for crystal determination from XRD pattern, implemented in MS modelling Ver 8.0 (Accelrys Inc.) was employed. Peak broadening (pseudo-Voigt function), asymmetry correction (Berrar–Baldinozzi function), zero-shift errors and lattice parameters were refined together to achieve the improved profile fitting. The refinement converged to Rwp = 5.07%, Rp =3.90% for a slipping AA stacking pattern.

Quantum simulation.

Theoretical calculation was carried out by using the plane-wave technique as implemented in CASTEP code. A 800 eV cutoff energy was used for the plane-wave basis set, and norm conserving pseudopotentials were employed for all ions. The exchange correlation energy is described by the generalized gradient approximation (GGA) in the form of Perdew, Burke, and Ernzerhof (PBE). To eliminate the interlayer interaction, single-layer Cu-BHS is simulated by introducing a vacuum layer larger than 15 Å.

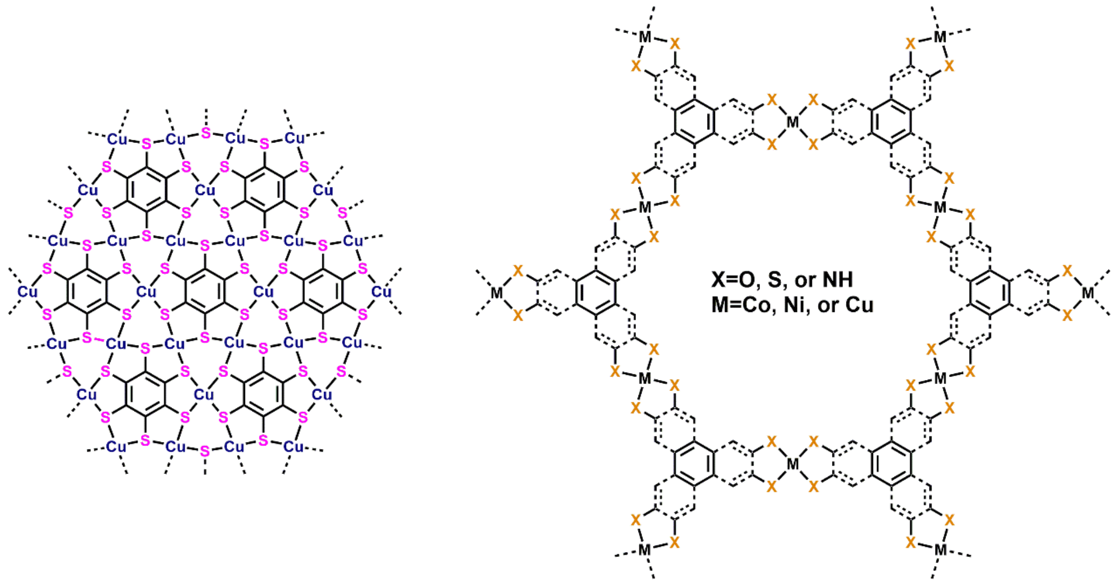

Figure S1 Structure of Cu-BHT (left) and other typical π-d conjugated CPs based on BHT and its analogues (right).

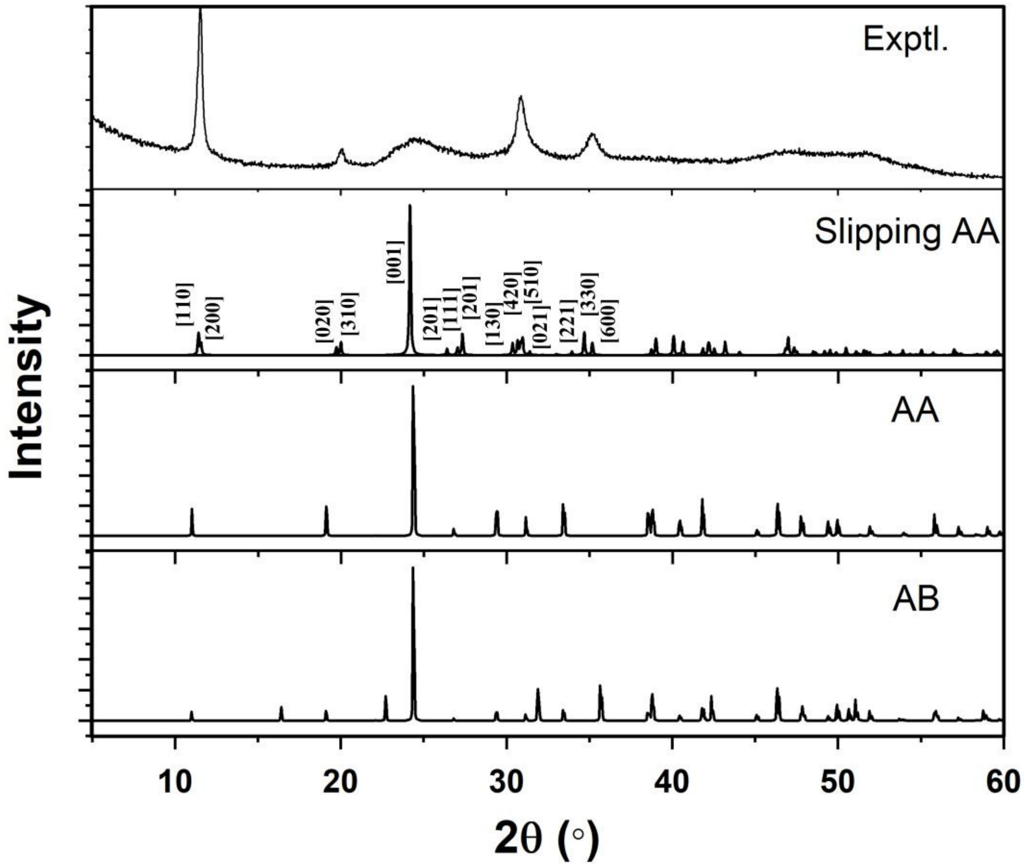

Figure S2. A comparison of the PXRD of the Cu-BHS and simulated patterns of structure models with slipping AA, AA and AB stacking patterns.

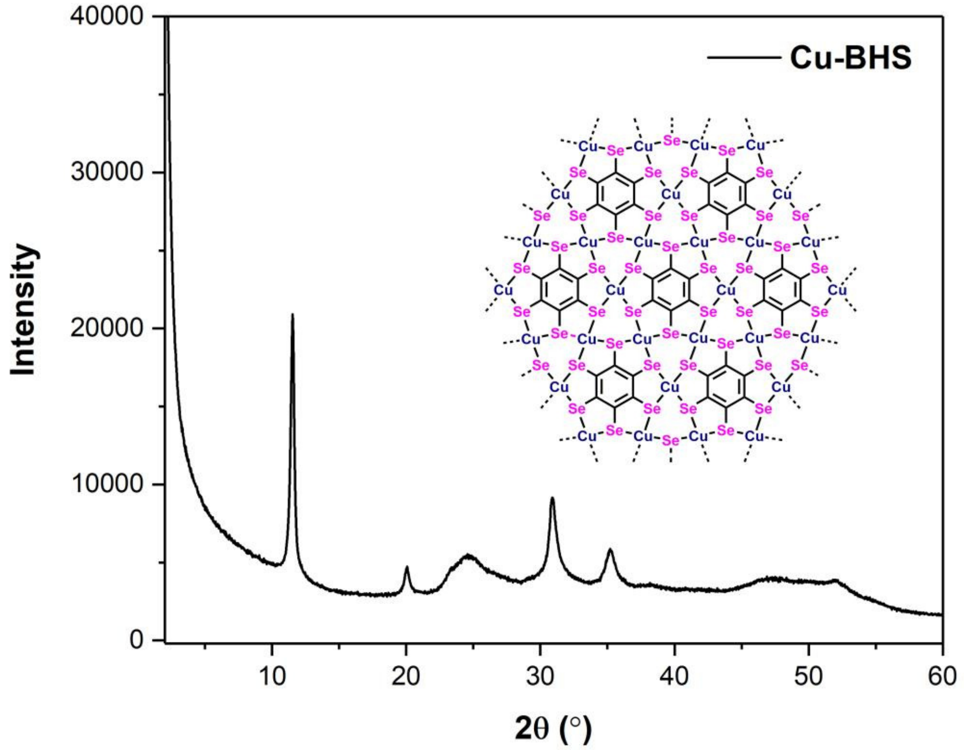

Figure S3. PXRD of the Cu-BHS (2θ=2-60°).

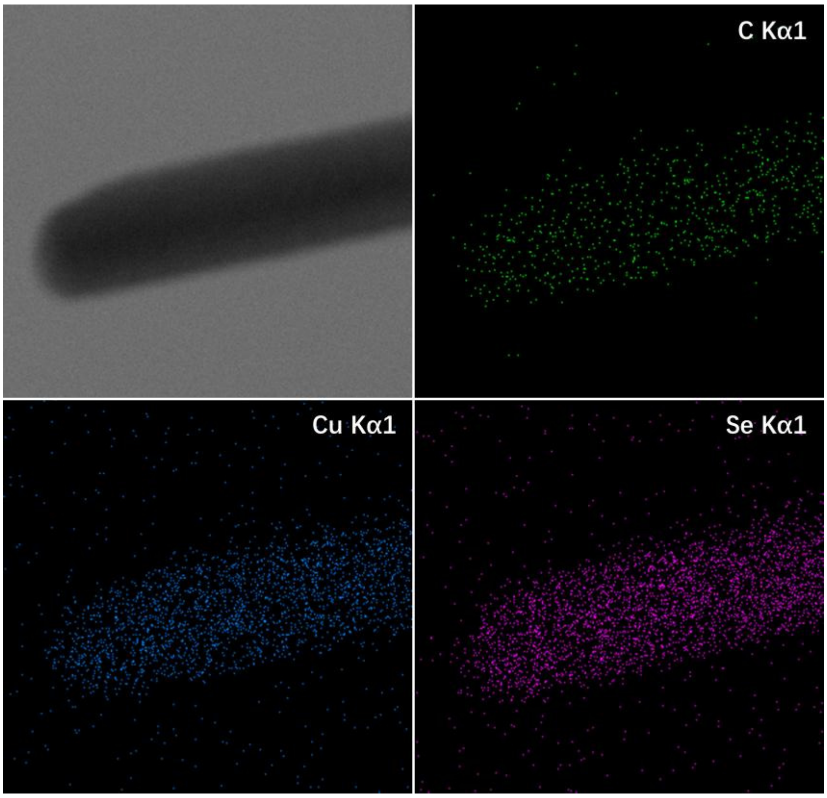

Figure S4. Elemental mapping of Cu-BHS nanocrystal.

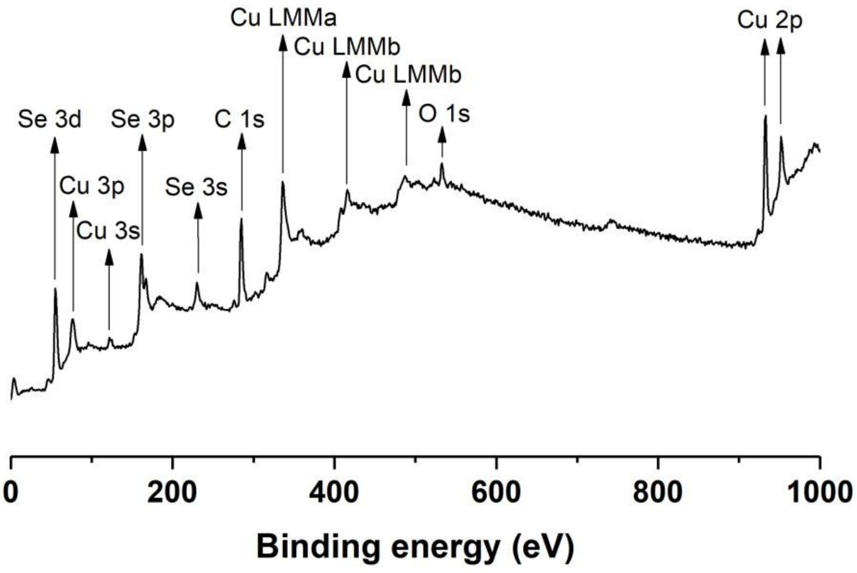

Figure S5. XPS full spectrum of Cu-BHS.

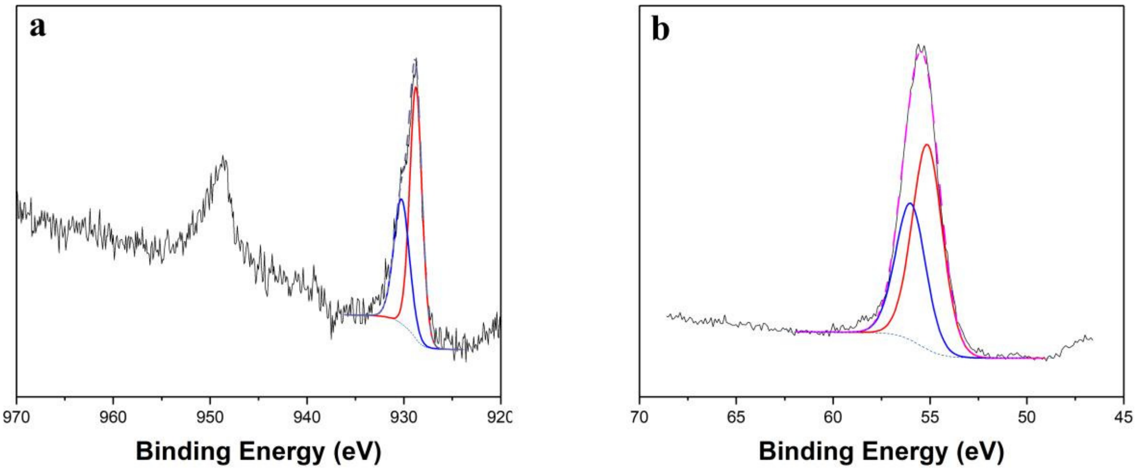

Figure S6. XPS spectra analysis of Cu-BHS (a) Cu 2p (b) Se 3d.

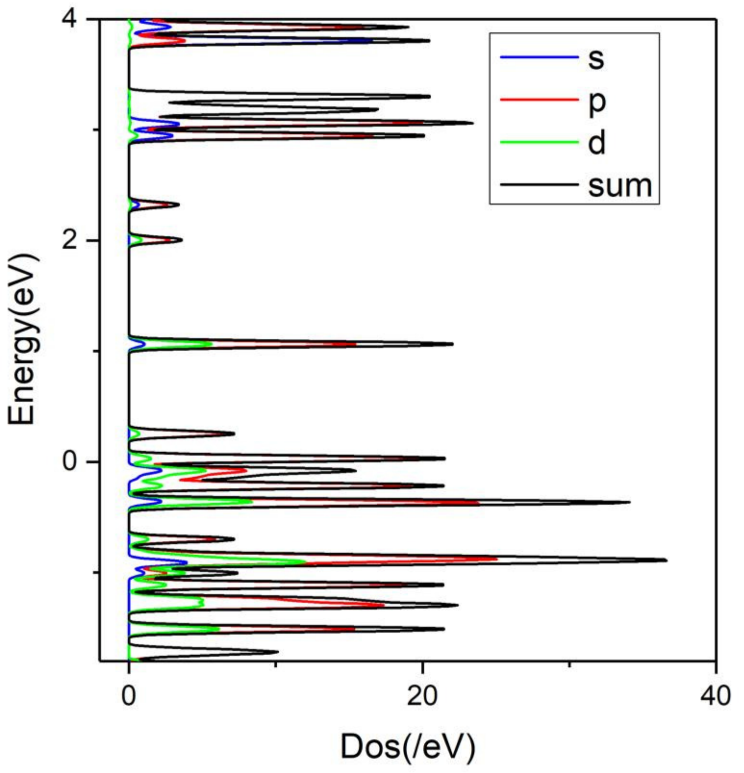

Figure S7. Density of State (DOS) of the monolayer of Cu-BHS complex calculated at PBE level.

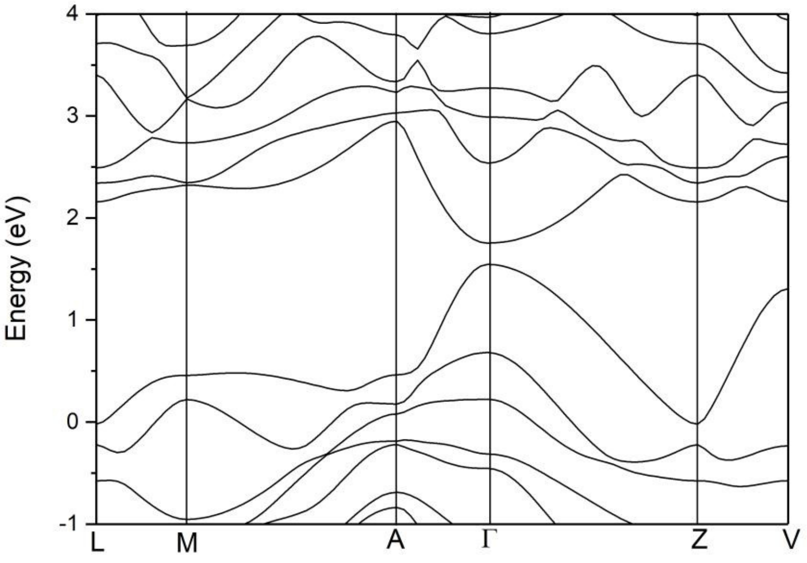

Figure S8. Band structure of the sliping AA stacking model of Cu-BHS complex calculated at PBE level. K point Γ=(0,0,0); A=(-0.500,0.000,0.000); M=(-0.500,-0.500,0.500); L=(-0.500,0.000,0.500); Z=(-0.000,-0.500,0.500); V=(0.000,0.000,0.500). The Fermi level is at zero.

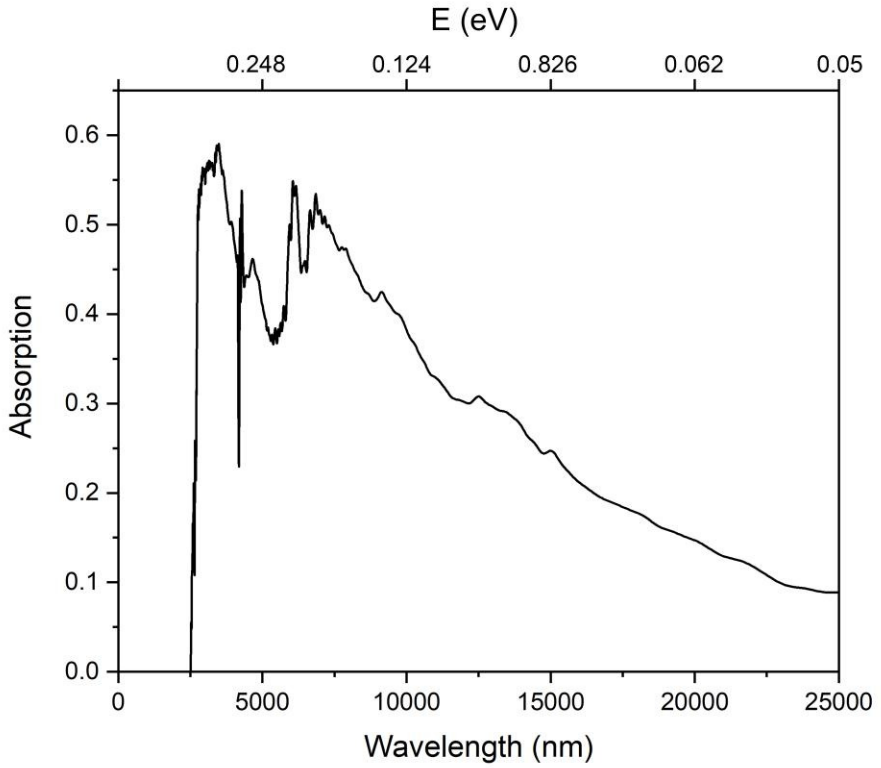

Figure S9. Infrared absorption spectrum of Cu-BHS.

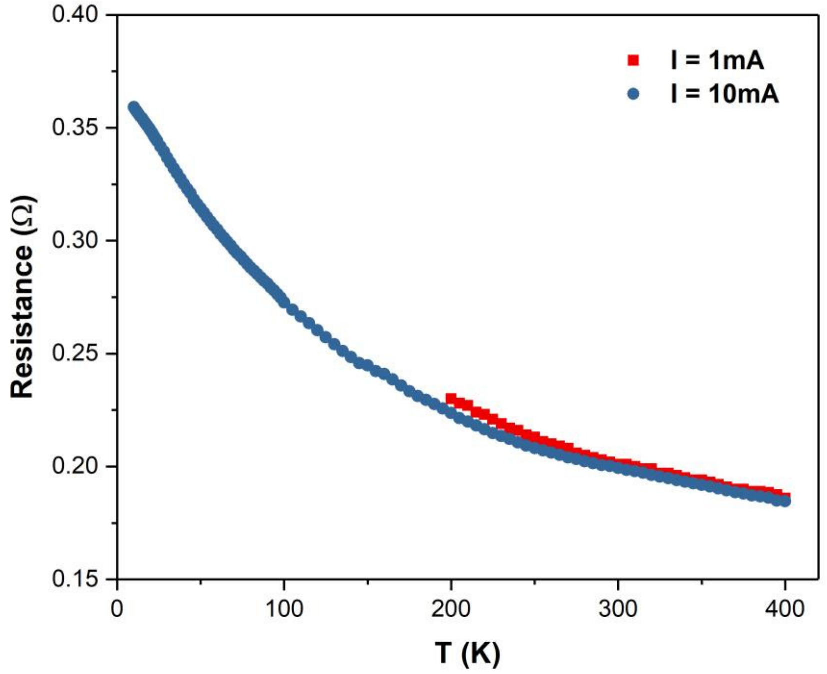

Figure S10. The temperature dependence of resistance measured under two different electric current (1mA and 10mA).

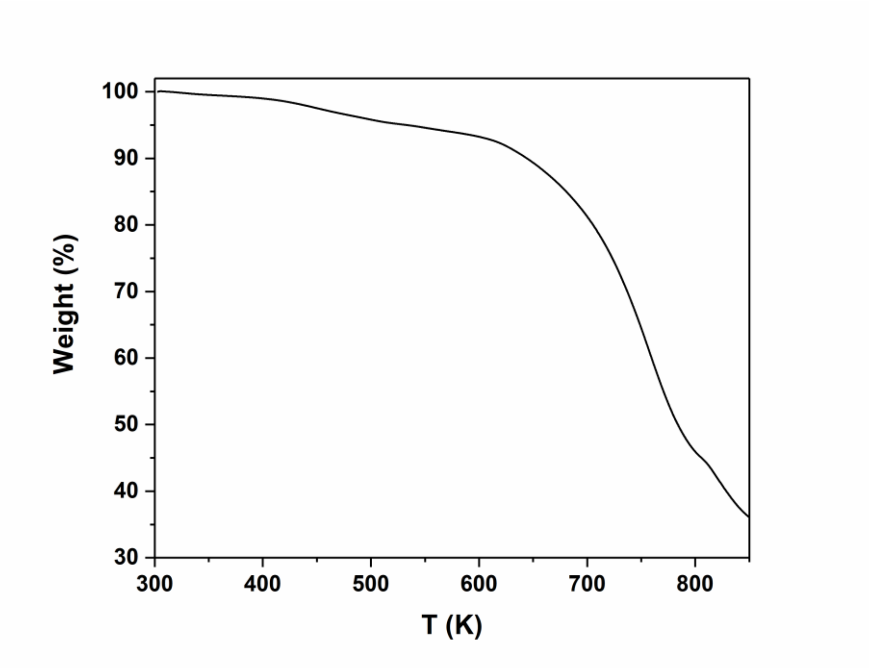

Figure S11. TGA analysis of Cu-BHS. The polymers begin marked weight loss at 420 K.

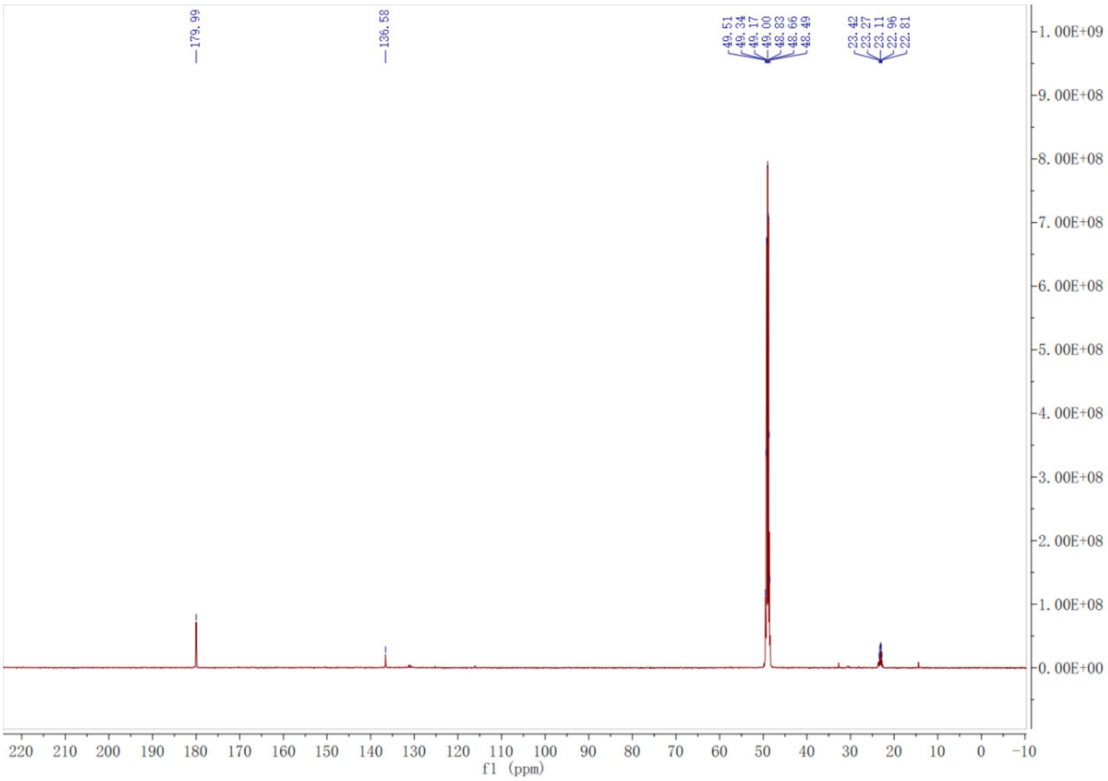

Figure S12. ^{13}C-NMR spectra measured on a solution which was obtained by dispersing BHS(BBr)$_3$ in AcONa-d3/MeOH-d4 (~5%, w/w). The distinct peak at 136.5 ppm correspond to the sp2 carbon of benzene ring in BHS fragment, the single peak at 179.99 ppm and the five peaks at ~23.11 ppm originate from AcONa-d3, and the seven peaks at ~49.00 ppm originate from MeOH-d4.

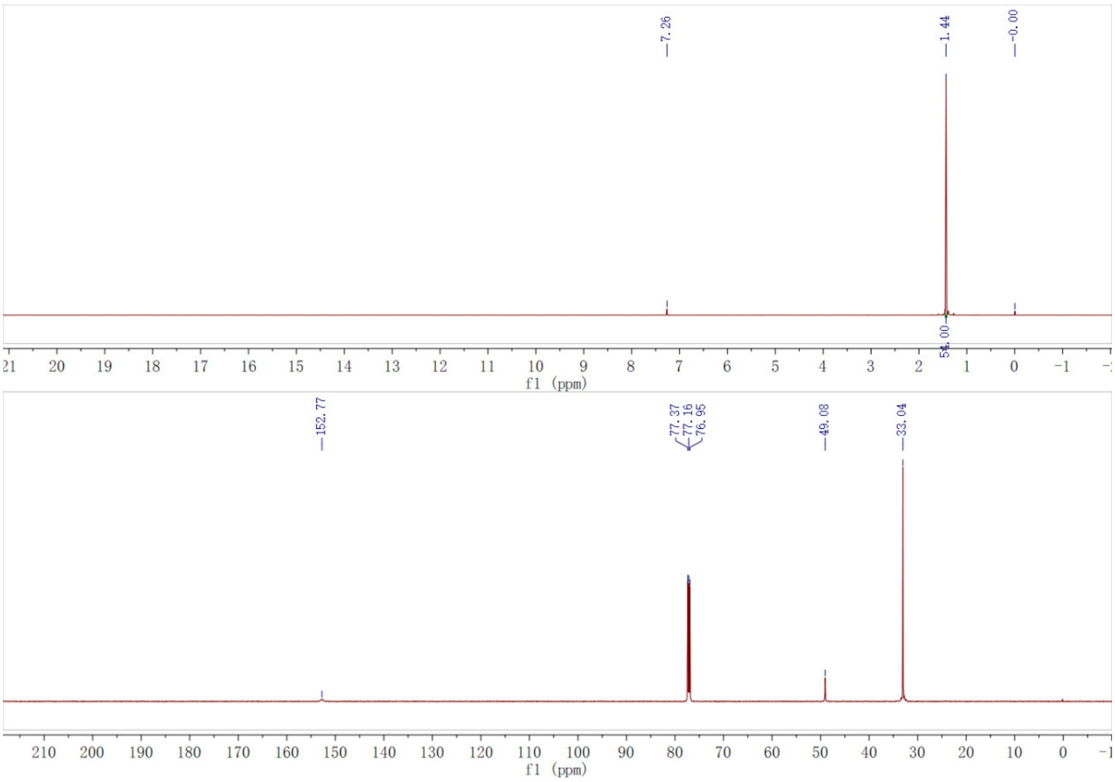

Figure S13. ^{1}H-NMR and ^{13}C-NMR spectra of t-Bu_6BHS.

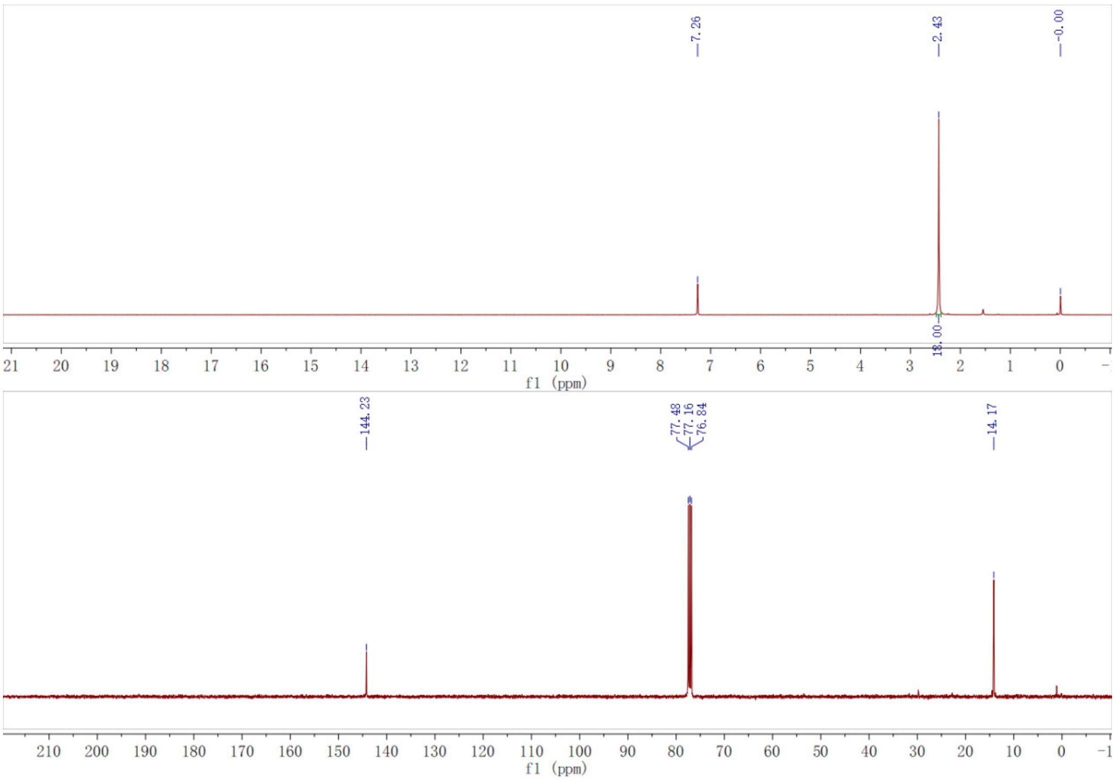

Figure S14. ^{1}H-NMR and ^{13}C-NMR spectra of Me_6BHS.

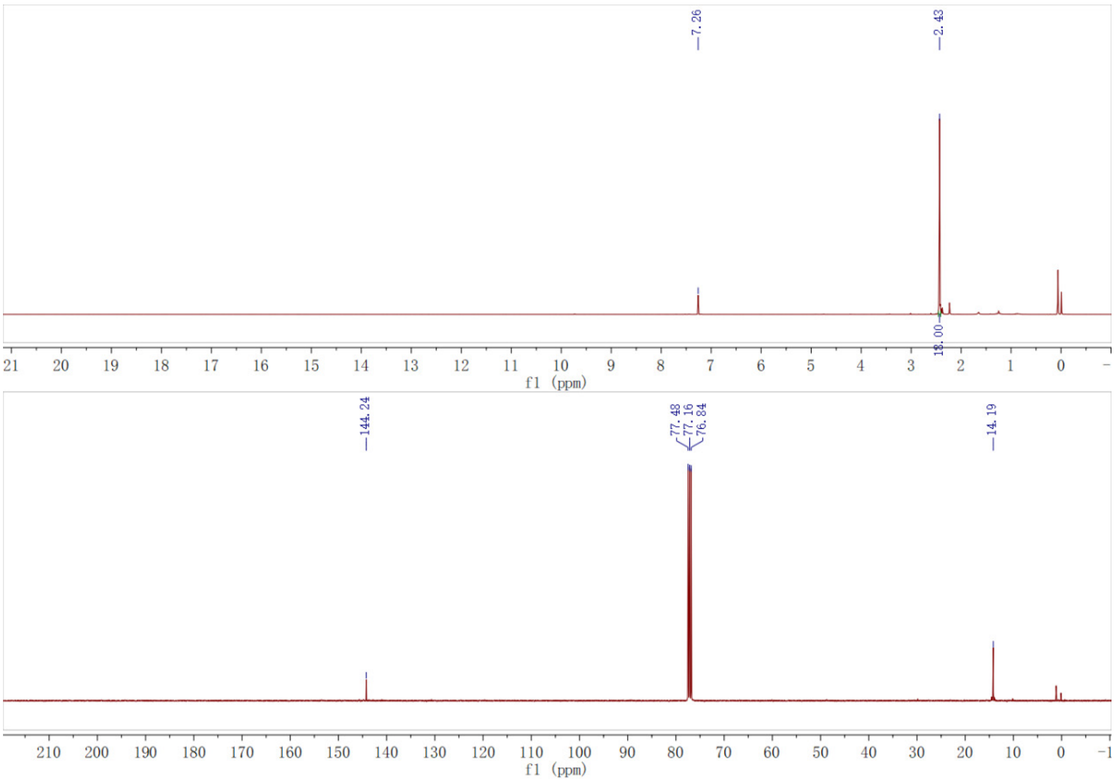

Figure S15. ^{1}H-NMR and ^{13}C-NMR spectra of Me_6BHS (crude product obtained via method 1).

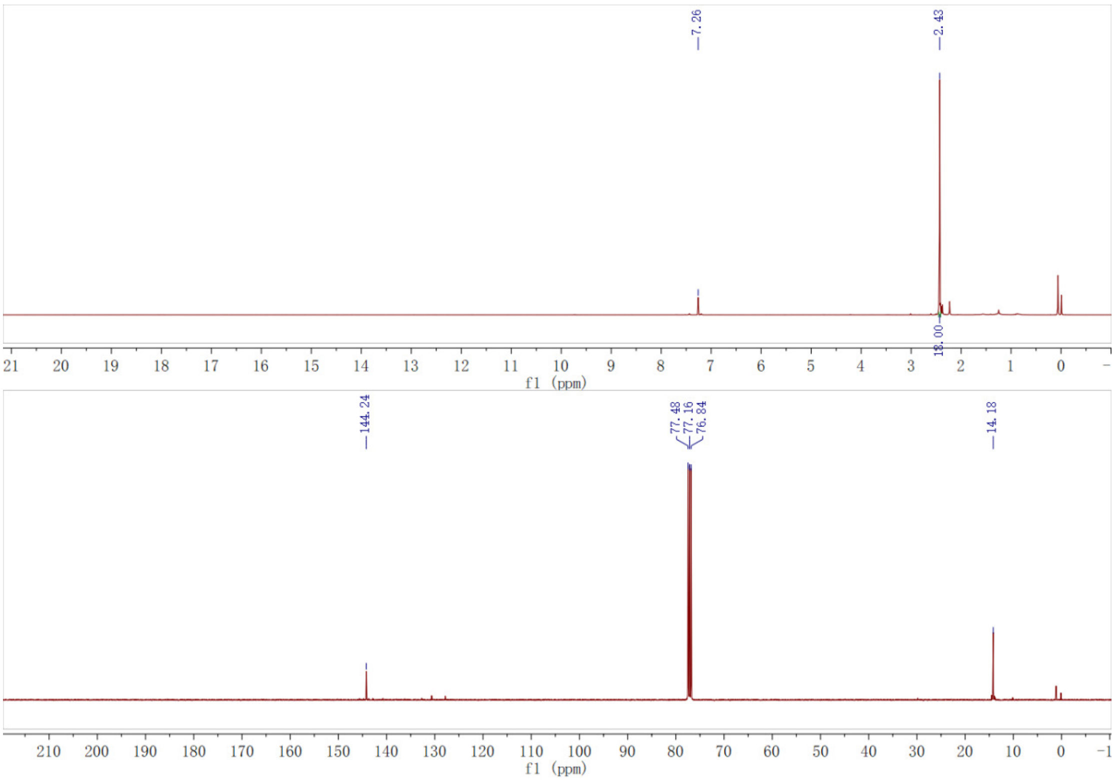

Figure S16. ^{1}H-NMR and ^{13}C-NMR spectra of Me_6BHS (crude product obtained via method 2, in the presence of BH_3).

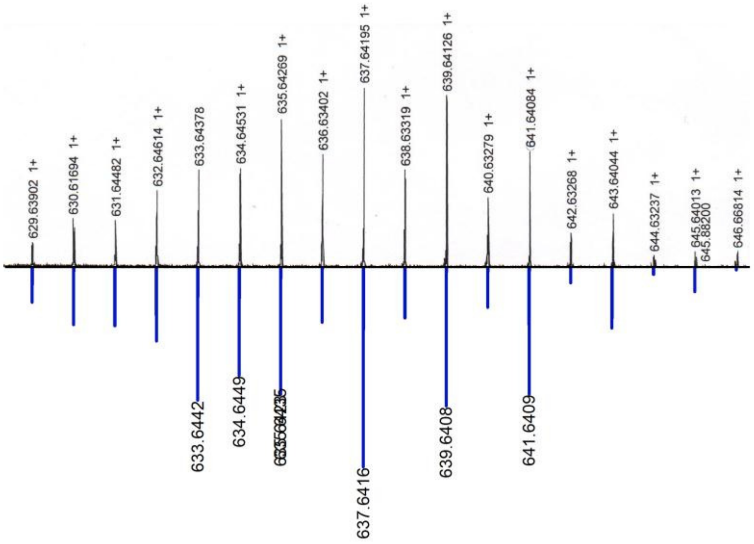

Figure S17. High resolution mass spectrum (HR-MS) comparison of experimental result (black lines) with the calculated result from $[C_{12}H_{18}Se_6]^+$ (blue lines).
